# Exploration of exposure to artificial intelligence in undergraduate medical education: a Canadian cross-sectional mixed-methods study

**DOI:** 10.1186/s12909-022-03896-5

**Published:** 2022-11-28

**Authors:** Aidan Pucchio, Raahulan Rathagirishnan, Natasha Caton, Peter J. Gariscsak, Joshua Del Papa, Jacqueline Justino Nabhen, Vicky Vo, Wonjae Lee, Fabio Y. Moraes

**Affiliations:** 1 School of Medicine, Queen’s University, 15 Arch Street Kingston, Kingston, ON K7L 3N6 Canada; 2 Department of Medicine, University of British Columbia, 317 - 2194 Health Sciences Mall, Vancouver, BC V6T 1Z3 Canada; 3 School of Medicine, Federal University of Paraná, Rua XV de Novembro, 1299 - Centro, Curitiba, PR 80060-000 Brazil; 4 Schulich School of Medicine & Dentistry, London, Ontario Canada Schulich School of Medicine & Dentistry, Western University, Clinical Skills Building, London, ON N6A 5C1 Canada; 5 Michael G. DeGroote School of Medicine, McMaster University, 1280 Main Street West, Michael DeGroote Centre for Learning and Discovery – 3104, Hamilton, ON L8S 4K1 Canada; 6 Department of Oncology, Queen’s University, 25 King St W, Kingston, ON K7L 5P9 Canada; 7 Kingston Health Sciences Centre, 25 King St W, Kingston, ON K7L 5P9 Canada

**Keywords:** Artificial intelligence, Curriculum, Deep learning, Education, medical, Machine intelligence, Machine learning, Undergraduate

## Abstract

**Background:**

Emerging artificial intelligence (AI) technologies have diverse applications in medicine. As AI tools advance towards clinical implementation, skills in how to use and interpret AI in a healthcare setting could become integral for physicians. This study examines undergraduate medical students’ perceptions of AI, educational opportunities about of AI in medicine, and the desired medium for AI curriculum delivery.

**Methods:**

A 32 question survey for undergraduate medical students was distributed from May–October 2021 to students to all 17 Canadian medical schools. The survey assessed the currently available learning opportunities about AI, the perceived need for learning opportunities about AI, and barriers to educating about AI in medicine. Interviews were conducted with participants to provide narrative context to survey responses. Likert scale survey questions were scored from 1 (disagree) to 5 (agree). Interview transcripts were analyzed using qualitative thematic analysis.

**Results:**

We received 486 responses from 17 of 17 medical schools (roughly 5% of Canadian undergraduate medical students). The mean age of respondents was 25.34, with 45% being in their first year of medical school, 27% in their 2nd year, 15% in their 3rd year, and 10% in their 4th year. Respondents agreed that AI applications in medicine would become common in the future (94% agree) and would improve medicine (84% agree Further, respondents agreed that they would need to use and understand AI during their medical careers (73% agree; 68% agree), and that AI should be formally taught in medical education (67% agree). In contrast, a significant number of participants indicated that they did not have any formal educational opportunities about AI (85% disagree) and that AI-related learning opportunities were inadequate (74% disagree). Interviews with 18 students were conducted. Emerging themes from the interviews were a lack of formal education opportunities and non-AI content taking priority in the curriculum.

**Conclusion:**

A lack of educational opportunities about AI in medicine were identified across Canada in the participating students. As AI tools are currently progressing towards clinical implementation and there is currently a lack of educational opportunities about AI in medicine, AI should be considered for inclusion in formal medical curriculum.

**Supplementary Information:**

The online version contains supplementary material available at 10.1186/s12909-022-03896-5.

## Background

It is likely that artificial intelligence (AI) technologies will be incorporated into clinical practise, with applications such as image-based diagnostics in radiology demonstrating accuracy and efficacy rivalling speciality trained physicians [1–2]. While many of these technologies remain investigational, the traditional roles of physicians, particularly those who perform imaging-based diagnostics, are expected to change in some capacity as AI tools progress towards clinical implementation [3, 4]. Concrete skills about how to interpret and use AI and integrate AI tools into clinical workflow may become important for physicians in the near future [3]. Additionally, other key skills in medicine such as the ethical aspects of decision making or humanization and empathy through the doctor–patient interaction may become more important [3–5]. Professional and regulatory bodies have also begun to recognize the value of AI as a core competency for physicians, as is demonstrated by the recent establishment of the Canadian Royal College of Physicians and Surgeons Task Force Report on Artificial Intelligence and Emerging Digital Technologies [6].

Despite the paradigm shift that AI may bring, there have been few developments in formal educational opportunities about AI or machine learning (ML) for medical trainees at all levels [3, 4]. Educational opportunities about AI and ML for physicians and medical learners remain optional, inconsistent between institutions, and largely focus on research applications [3]. A lack of knowledge about AI and it’s uses in a clinical context will plausibly pose a barrier to future uptake and effective use among physicians [3, 4]. Previous studies have addressed the perceptions of undergraduate medical students about AI in medicine, noting that they believe AI will be an integral part of medical practise in the future [7, 8]. These studies have also found poor self-reported knowledge about AI among medical trainees, including undergraduate medical students [7, 8]. However, there have been no studies to date that identify and assess exposure to existing educational opportunities about AI in medicine among undergraduate medical students, or that gauge the interest and perceived need for AI education among these learners [7, 8]. Additionally, the AI content that students would be receptive to learning about and the desired mediums of curriculum delivery have not been identified. This data could strengthen existing educational opportunities, support the inclusion of AI in formal medical curriculum, and ensure that AI curriculum is well received by undergraduate medical learners.

To address this knowledge gap, we performed a national survey among all undergraduate medical students in Canada. The survey aimed to assess undergraduate medical students’ feelings about AI in medicine, identify the currently available educational opportunities about AI in medicine, explore the perceived need for AI inclusion in medical curriculum, and identify desired mediums for AI curriculum delivery. Given the heterogeneity of education about AI in medicine, follow-up interviews were performed with a sample of participants to provide further insight into educational opportunities about AI that were not captured using the survey instrument.

## Methods

This cross-sectional mixed methods study had both an online survey and interview component. A mixed-methods approach was selected as the survey component enables data collection from a large quantity of respondents at all Canadian medical schools, while the interview component allows for a more holistic exploration of education of AI in medicine and provides narrative context. Given how variable curriculum can be between institutions, interviews are particularly valuable in exploring institutionally specific opportunities or deficits that cannot be captured by the survey instrument. The survey was distributed to all 17 Canadian medical schools through various digital mediums including social media, student portal, and undergraduate medical newsletters, as dictated by the institutional requirements (Additional file 1). A random sample of participants who opted-in to interviews during the survey (responded “Yes” to the question “Would you be willing to provide a short, recorded interview about your responses at a later date?”) were contacted to participate in interviews. Institutional ethics approval was obtained by the Health Sciences and Affiliated Teaching Hospitals Research Ethics Board at Queen’s University (ID# 6031912).

### Survey design

A 56 question survey was developed in accordance with the Consensus-Based Checklist for Reporting of Survey Studies (CROSS) guidelines for survey studies and coded in Microsoft Forms (Additional file 2 [9];). The CROSS guidelines are a well established framework for developing and reporting survey based studies. The survey was available in both English and French. The survey was piloted by medical students on the research team that were not involved in survey instrument creation to ensure question clarity and accessibility of Microsoft Forms (PG, JDP, VV, WL); no formal pretest was performed. Assessment of inclusion eligibility occurred at the start of the questionnaire, with inclusion criteria being consent to participate and enrollment in Canadian medical school at any time in 2021. Participation was entirely voluntary. A chance to win one of four $50 gift cards was offered as incentive for participation. Survey distribution occurred from May 2021 to October 2021. Each institution was given a 1 month period to respond to the survey after initial distribution, with a reminder issued with 2 weeks remaining. While it is impossible to know how many students received the survey given the variable methods of distribution, it is plausible that all of the ~ 10,000 Canadian undergraduate medical students had the opportunity to respond [10]. All responses were anonymous to the research team, although emails addresses were collected to contact participants for interviews and to prevent multiple responses from participants.

The first section of the survey contained six screening questions exclude participants who did not meet inclusion criteria, and logistical questions regarding the gift card draw and the participants willingness to participate in the interview portion. The second section of the survey contained 12 questions identifying participant demographics. The third section of the survey consisted of five questions about the participants knowledge of AI in daily life. The fourth section consisted of 20 questions about the participants attitudes, beliefs, and knowledge regarding AI in medicine, including 16 Likert scale questions scored from 1 (strongly disagree) to 5 (strongly agree). The final section consisted of 13 questions (12 Likert scale) about participant access to educational opportunities about AI during their medical training and their preferred formats to learn about AI.

### Interview design

The interview study component was developed in accordance with the Consolidated Criteria for Reporting Qualitative Research (COREQ) guidelines for qualitative research reporting [11]. Participants attended a 10–15 minute interview with one interviewer via video conference (Zoom Video Communications, San Jose, California) between October 2021 to December 2021, after the closure of the survey. The interview consisted of six scripted interview questions that required participants to use retrospective recall to recount their experiences and feelings about their previous experience with AI in medical school and their thoughts about receiving AI education during their medical training (Additional file 3 [9, 10 12,13];). The purpose of the additional interview component was to inform future research and the development of curriculum about AI in healthcare. The interview audio was recorded and transcribed verbatim digitally for analysis.

#### Selection of participants

In an effort to interview participants from each of the English speaking medical schools (14 of the 17 medical schools), a stratified random sampling method was employed. A random number generator was used to select two participants from each school, who were then invited to interview. A follow-up email was sent if the participant did not respond in 1 week. If a participant failed to respond to their invitation in 2 weeks, another participant was randomly selected using the same method and invited. There were no specific inclusion/exclusion criteria and participation was voluntary. All participants were originally contacted via email by a member of the research team (AP, NC). There were no additional incentives or financial compensation were offered for participation.

#### Characteristics of study subjects

Seventeen undergraduate medical students were recruited from 11 medical schools. Nine participants were female and eight were male. Three participants were currently in their first year of medical school (MS1), seven were MS2, three were MS3, two were MS4, and two had most recently completed MS2 and were on leave to complete the PhD component of their MD PhD. The average age of the participants was 26.1 (range of 23–30). All participants were familiar with the research goals. No participants declined to interview or dropped-out from interviews, although three students were invited to interview and did not respond.

#### Protocol for responses

Two interviewers performed the interviews (AP, NC), and audio from the video interviews was recorded. At the start of each interview the study goals were explained, and verbal consent was obtained. No follow-up questions were asked, the interviewers provided no additional information during the interviews, and there was no repeat questioning at a later date. Nobody else was present during the interview. Survey transcripts were not returned to participants for comment or correction.

#### Research team and reflexivity

Project conception and survey design were performed by AP, RR, NC, JJN, FYM. AP is a male medical student with a research background in medical education, AI, and ophthalmology. RR is a male medical student with a research background in medical education, plastic surgery, and qualitive improvement. NC is a female medical student who holds an MPH and has research experience in public health. JJN is a Brazilian female medical student who has research experience in medical education and AI. FYM is a staff radiation oncologist who holds a PhD in radiation oncology. FYM has extensive research experience in oncology and AI. Two of the interview participants were personally known to AP and RR, while all participants were unfamiliar to the second interviewer and the rest of the research design team (NC, FYM). Members of the interview and research team had previously expressed that they believe AI training in medicine in important in their published work [3].

### Analysis

Statistics were performed in SPSS 27.0 (IBM Corp., Armonk, NY). Quantitative demographic data were reported descriptively, as a count and a percentage. Likert scale data were reported as the percentage of participants that either agreed or disagreed with each given statement, and the count of relevant responses. Each of the statements in the results is based on a single Likert scale question. Participants who failed the screening questions or did not provide responses to any of the questions were removed from the data. Box plot figures of Likert scale data were created using R (R Core Team, Vienna, Austria).

The text transcriptions of interview responses were analyzed sentence by sentence using emergent thematic analysis to explore the experiences of participants with AI education in medicine and their perceived need for AI education [14]. Qualitative analysis software (NVIVO 12 Pro, QSR International, Melbourne, Australia) was used to organize, sort, and code the data. Two independent, blinded coders (AP, NC) performed emergent coding consisting of assigning all phrases of the interview data into codes, regardless of their expected relevance to future themes [14, 15]. Codes were initially developed as the smallest unit of analysis, with similar codes being grouped together to form subthemes, and subthemes further grouped together to form themes. The process of theme development was iterative, with frequent revisions as patterns became apparent. Finalization of themes occurred after review and discussion by the two coders. Emerging patterns were noted based on coding frequencies. No coding diary was maintained during the analysis process, with no formal comment on bias or emerging themes; instead email communication and regular meetings between team members supported accurate, unbiased coding, ameliorating personal biases or preconceptions. The inter-coder reliability was 82%.

## Results

### Survey (quantitative analysis)

In total, 486 responses were obtained and 475 met the inclusion criteria, with survey respondent demographics outlined in Table 1. The following data is from survey sections 1–3. Notably, 273 (57.3%) respondents indicated having a family member with an advanced degree, 183 (38.5%) considered themselves to have a high degree of technological literacy, and 74 (15.6%) had a background in mathematics, statistics, or computer science. Many students reported being aware of the use of AI in medicine (83.8%, 398), with the most common sources of information being peers in healthcare (23.8%, 200), peers outside of healthcare (16.6%, 140), and published papers (16.1%, 135). Characteristics regarding sources of AI education are detailed in Table 2. The majority of students (75.8%, 360) reported that they had received no formal teaching about AI during medical school. The preferred learning format about AI in medicine were workshops (27.1%, 299), lectures (23.8%, 263), and collaborative activities with other departments such as computer science (17.3%, 191). All survey Likert scale results are summarized in Additional file 4.

**Table 1:** Survey demographic data

Age, mean (SD)	25.34 (4.26)
Total participants	475
**Survey Population Characteristics**	**N (%)**
Gender	
Female	300 (63.2)
Male	166 (34.9)
Non-binary	3 (0.6)
Prefer not to disclose	5 (1.1)
Year of Medical Training	
1st	214 (45.1)
2nd	130 (27.4)
3rd	73 (15.4)
4th	48 (10.1)
M.D. MSc	4 (0.8)
M.D. PhD	5 (1.1)
What level of education did you achieve before beginning medical education?	
High School	18 (3.8)
College Diploma	33 (6.9)
Bachelor’s Degree	287 (60.4)
Master’s Degree	120 (25.3)
PhD or Doctorate	13 (2.7)
Prefer not to disclose	3 (0.6)
Do you have an immediate family member with an advanced degree (Master’s or Doctorate or similar)?	273 (57.5)
Do you have a background in mathematics, statistics, or computer science?	74 (15.6)
Do you consider yourself to be “tech-savvy”, or have a high degree of technological literacy?	183 (38.5)

**Table 2:** Survey population characteristics

	N (%)
**Many applications we use in daily life use artificial intelligence. Were you aware of this?**	***N = 475***
Yes	263 (55.4)
Yes, but superficially	181 (38.1)
No	31 (6.5)
**If yes, where have you learned about uses of artificial intelligence in daily life?**	***N = 1250***
Formal education (University, College)	115 (9.2)
Work experience	132 (10.6)
Scientific literature	144 (11.5)
News	256 (20.5)
Social media	285 (22.8)
Lectures	98 (7.8)
Friends/family	200 (16.0)
Other	20 (1.6)
**Have you learned about artificial intelligence in formal education prior to, or during, medical school?**	***N = 475***
Yes	115 (24.2)
No	360 (75.8)
**If yes, where in your medical education or prior education did you learn about artificial intelligence?**	***N = 168***
As a formal part of my medical degree curriculum	8 (4.8)
As part of an optional course offered by a medical school	4 (2.4)
As an online course	18 (10.7)
As a part of a research project	33 (19.6)
As part of an advanced degree (Master’s or higher)	34 (20.2)
As part of my undergraduate degree	63 (37.5)
Other	8 (4.8)
**Are you aware that artificial intelligence is currently utilized in medicine?**	***N = 473***
Yes	153 (32.2)
Yes, but superficially	245 (51.6)
No	75 (15.8)
**If yes, where have you learned about uses of artificial intelligence in medicine?**	***N = 839***
Formal education during medical school	52 (6.2)
Formal education in an advanced degree	43 (5.1)
Formal education in an undergraduate degree	54 (6.4)
My peers (healthcare)	200 (23.8)
My friends (non-healthcare)	140 (16.6)
My mentors/teachers (clinical training)	130 (15.5)
Published papers	135 (16.1)
The media	16 (1.9)
Movies and TV series	16 (1.9)
Other	53 (6.3)
**My preferred format for learning about artificial intelligence in medicine is:**	***N = 1105***
Lectures	263 (23.8)
Workshops	299 (27.1)
Conferences	165 (14.9)
Extracurricular activities	161 (14.6)
Collaborative activities with other departments (mathematics, computer science)	191 (17.3)
Other	26 (2.4)

#### Understanding Artificial intelligence and machine learning in medicine (survey section 4, Fig. 1)

Only 39% (34 strongly agree, 148 agree) of respondents were able to describe AI, machine learning, neural networks, and deep learning and 63% did not understand AI research methods. Student perceptions of AI in medicine included: the belief that AI has improved medicine (74% agree; 102 strongly agree, 234 agree), that AI is commonly used in medicine (*M*59% agree; 54 strongly agree, 217 agree), and that AI will revolutionize medicine in the future (74% agree; 150 strongly agree, 186 agree). Respondents agreed that artificial intelligence will be cost-effective (64%; 96 strongly agree, 199 agree) and optimize physician’s work (77% agree; 96 strongly agree, 256 agree), however students did not believe that some or all physicians would be replaced by AI (66% disagree; 121 strongly disagree, 181 disagree) and were not frightened by the development of AI (53% disagree; 62 strongly disagree, 182 disagree). Medical students were unsure if AI would particularly affect their specialty of choice (31.7% agree, 35.1% disagree), but agreed that they will need to understand AI throughout their career (68.3% agree; 104 strongly agree, 212 agree) and that they would use applications of AI during their careers (72.9% agree; 110 strongly agree, 223 agree).

**Fig. 1:**
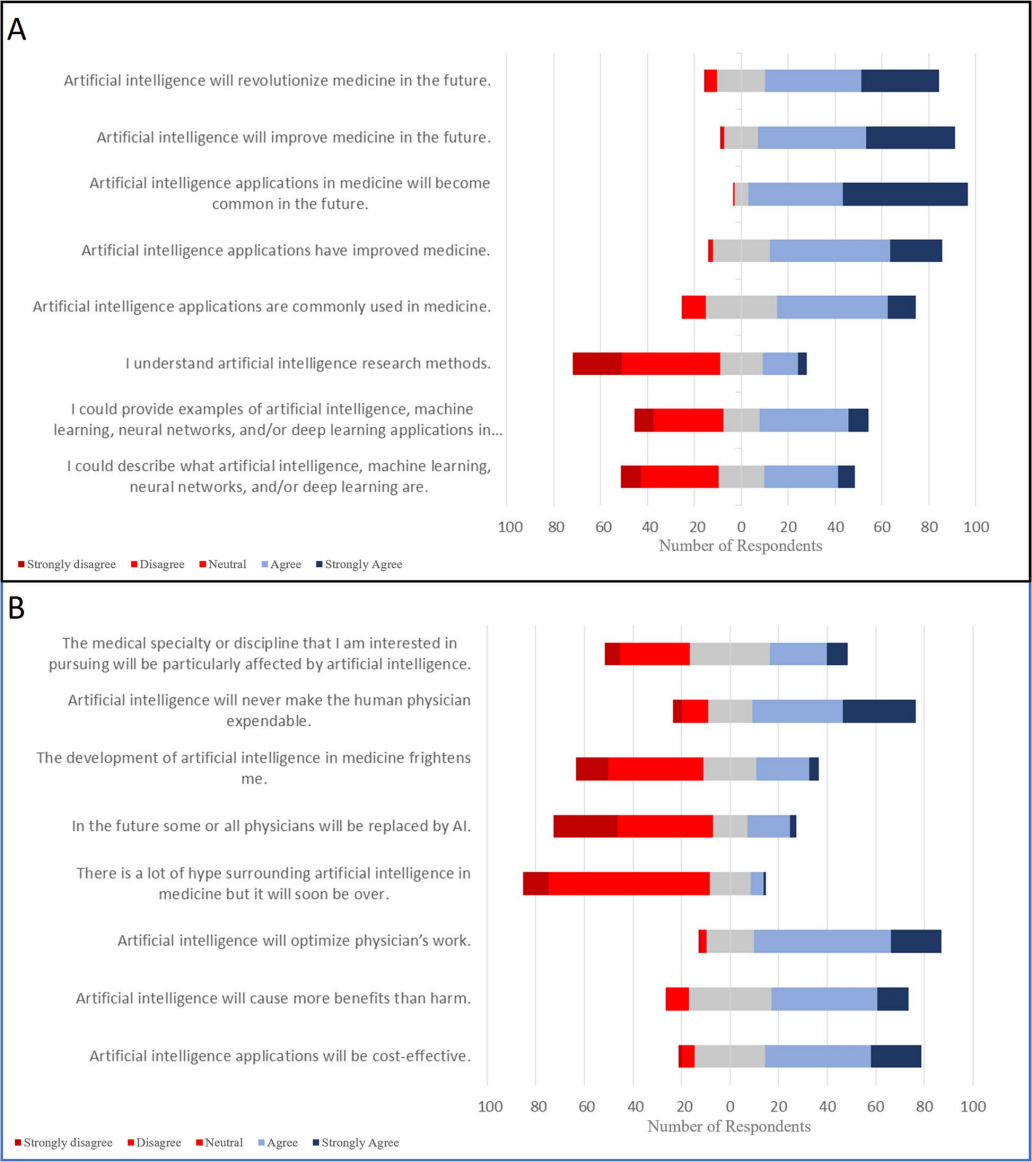
Participant Likert responses about AI in medicine. **A**. Knowledge about current uses of AI in medicine. **B**. Perceptions about the future of AI in medicine

#### Artificial intelligence in medical education (survey section 5, Fig. 2)

Respondents believed that AI should be formally taught in medical education (67% agree; 99 strongly agree, 205 agree), but indicated they had not received training in formal curriculum previously (85% disagree; 181 strongly disagree, 196 disagree). Medical students had not received training in AI through education external to formal medical school curriculum (66% disagree; 101 strongly disagree, 191 disagree), or research or work experiences (70% disagree; 122 strongly disagree, 197 disagree). Some students had independently learned about AI (41.9% agree, 42.4% disagree). Survey respondents disagreed that learning opportunities regarding AI in medicine have been adequate (74% disagree; 87 strongly disagree, 244 disagree). Students agreed that their understanding of programming or mathematics were a barrier to understanding AI (47% agree; 70 strongly agree, 141 agree). Students agreed that it is important to study AI in medicine (62% agree; 70 strongly agree, 214 agree) and that given the chance they would like to learn more about AI (78% agree; 150 strongly agree, 210 agree).

**Fig. 2:**
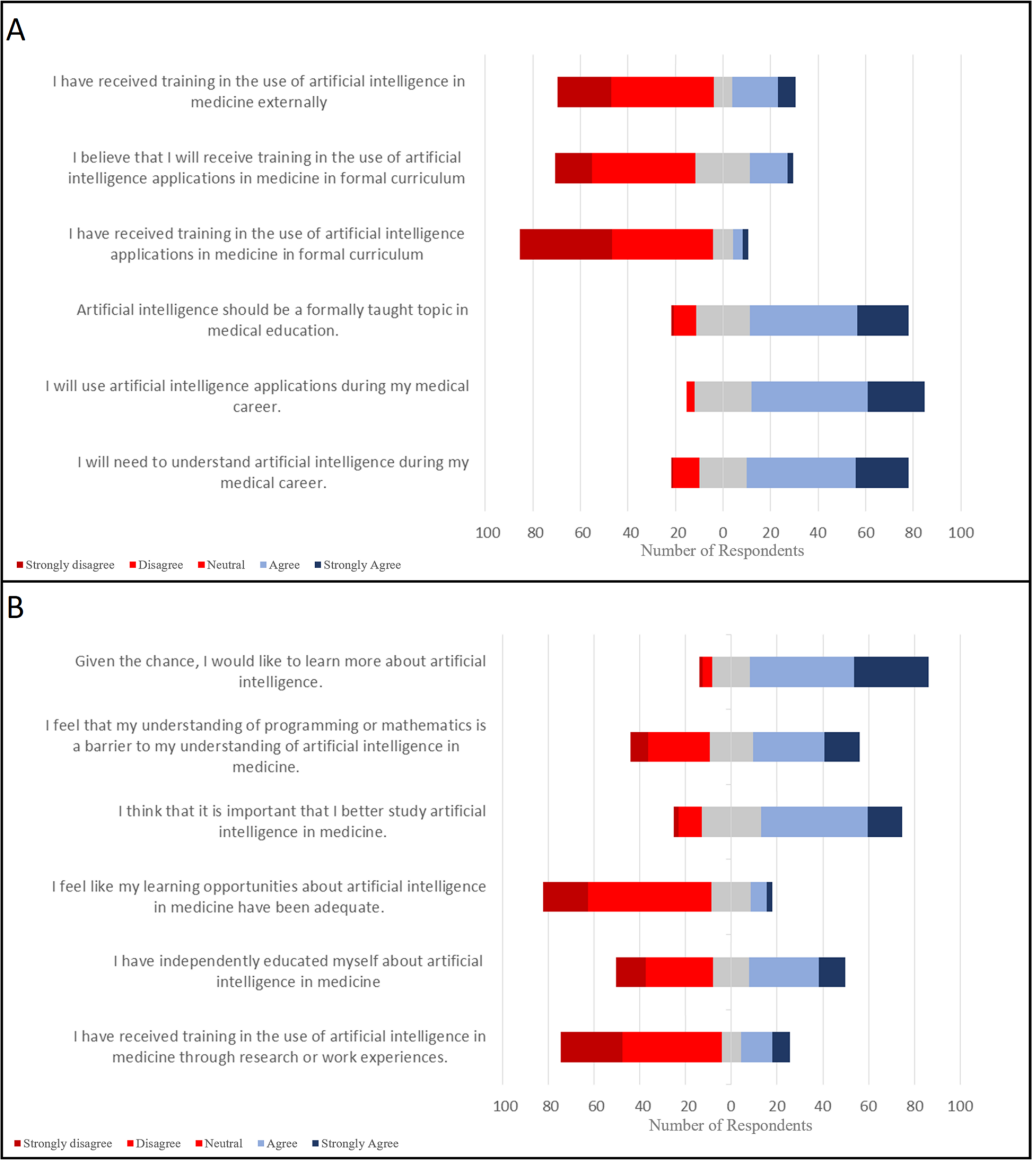
Participant Likert responses about the AI in medical education. **A**. Current educational opportunities about AI in medicine. **B**. Perceptions about the future of AI education in medicine

### Interviews (qualitative analysis)

Three major themes emerged in the qualitative analysis: a lack of existing learning opportunities about AI, the need to incorporate AI learning in medical curriculum, and positive sentiment about the future of AI in medicine. Subthemes that emerged included the value of elective or informal learning about AI, a scarcity of formal learning opportunities, the desired formats of future AI education, barriers to development of AI curriculum, excitement about the future of AI in medicine, fear about misuse or poor stewardship of AI, and specific uses for AI including use in specific disciplines.

#### Learning opportunities

The theme of existing learning opportunities about AI in medicine developed over many references across all participants. The vast majority of existing AI educational opportunities were elective or informal learning, with the most commonly identified opportunity being education prior to medical school or research, non-institutional, independent reading, and discussions with peers. One participant noted “When I was an undergrad, I did a course called ‘Engineering in Medicine’ and there was a big overview of the uses of AI and different types of image processing using AI.” Learning opportunities in education prior to medicine, particularly in interviewees who had done engineering degrees, or in research endeavors, were common; “… My first real introduction to AI in healthcare was when I was working as a research student the summer before I started medical school… we had a guest lecturer who came in and spoke about her work… it was an analysis of breast cancer pathology samples using AI and machine learning to do it a lot faster.” Non-institutional courses and workshops were also a common medium to learn about AI; “Outside of the curriculum there are a lot of student led workshops that teach fundamentals of artificial intelligence”.

All participants discussed formal institutional learning opportunities about AI, with all participants but one indicating that there were no formal educational opportunities about AI available to them. One student said “There’s no exposure [to AI in medical school]. I think we learn basic stats but nothing more,” a sentiment that was shared by almost all of the participants. Some quotes indicated that AI was referenced off-hand; “There hasn’t been anything in our formal curriculum… [AI] might have got mentioned in passing in one of our radiology lectures.” One MD/PhD candidate noted that some AI training was part of their postdoctoral training, while an undergraduate medical student noted that “The [University of Toronto] Center for AI Research and Education in Medicine has student representatives, so through them I’ve got some exposure….” No participants mentioned the inclusion of AI in formal undergraduate medicine lectures.

#### The need for medical curriculum about AI

Despite a lack of formal learning opportunities, the importance of learning about AI in medicine developed, was noted by the majority of participants. Many participants noted that “[learning about AI] is important, because I think that it’s going to be a reality in how a physician practices medicine and should be something we should learn,” while many references noted that “I don’t have a great understanding of what AI is capable of or what it even is. It definitely could have implications in different fields of healthcare, and I think we all need to be prepared in the future of medicine.” Students often discussed their desired learning formats and learning needs (82 references). While some students indicated that “… having like a lecture from engineers or computer scientists would probably be really helpful,” many believed smaller scale changes would also be useful; “I think just an addendum to an existing lecture… Even if it was just a few slides where AI is relevant to that field, I think it would be really helpful.” The idea of learning outside of traditional didactic lectures was also proposed, with one student suggesting “…something more accessible like a video or an audio podcast.”

While most participants did believe inclusion in curriculum was important, a small number of participants indicated that it should not be a priority. One student said “I think some people don’t really need to learn about AI or ML,” while another mentioned “I think that… my time would be better spent understanding the body and having [AI tools and results] interpreted for me by somebody who is an expert.” Barriers to inclusion of AI in medical curriculum was established by a small number of participants. Notably, many participants noted that AI shouldn’t take priority over other missing topics in curriculum. One participant said “I feel as though the preclerkship curriculum is already pretty packed with a lot of very relevant things… There are things I’m going to need to know as a clerk that I don’t feel like we’ve adequately covered.” Notably, no student had directly encountered this barrier, but rather it was speculated that curricular prioritization would be an issue. A lack of technical understanding of mathematics, programming, and computer science were also thought to be barriers to learning about AI, illustrated by one participant who joked: “I’m an old timer, I have issues with figuring out how to use my USB… I wouldn’t know where to start.”

#### Sentiment

Varying sentiments about AI were expressed by participants, with some references to excitement around the future of AI while other participants expressed concern or fear about AI or misuse of AI. Most of the positive references to the future of AI noted novel applications or the ability for technology to improve physician workflow. There were a variety of concerns brought up by participants, including “… concerns regarding the ethical issues surrounding AI” or the accuracy of AI tools. While infrequently noted, there was some concern about job security; “maybe there’ll be less jobs for physicians in certain fields that AI is more applicable to, like radiology or pathology.” There were many references to specific uses of AI, with imaging-based AI tools and specific professions such as radiology and pathology being the most commonly referenced subthemes. Some participants also thought there would be applications for patient use, such as “symptom checkers, [where the patient can] input the symptoms, and it spits out possible diagnoses.”

## Discussion

Our findings demonstrated that the majority of surveyed medical students believe that AI is important to the future of medicine and desire learning opportunities about AI. We also found that despite these attitudes, there remains a lack of educational opportunities across Canada at the institutions of study participants. With the rapid progression of AI tools towards clinical implementation and more prevalent use of AI in medical research, educational opportunities about AI need to be considered for inclusion in formal medical curriculum. Further, as the skillsets required to use AI may be different than those traditionally possessed by physicians, the desired learning formats, content interests, and perceived learning barriers of medical learners must inform the inclusion of AI content in medical curriculum.

Our findings are consistent with previous studies of medical learners, which have also identified limited knowledge of AI among medical trainees [7, 8, 16, 17]. A recent survey by Teng et al. (2022) found that medical students had limited knowledge about AI, suggesting that this indicated a need for urgent education. They noted that this growing knowledge gap would likely become a barrier to the development and use of AI in medicine, something that has been supported by other literature [1, 18]. Interestingly, this survey found that healthcare learners were optimistic of AI in their fields, although they were not sure it would be relevant in their field [7]. We also found a degree of cognitive dissonance among participants, as they believed that AI would revolutionize medicine while simultaneously believing AI would not directly affect them or their future practise. These findings could reflect poor understanding of AI applications, sensational reporting of AI in media or medical literature, or the limited exposure to AI in a clinical setting. Some recent survey cohorts of medical students have found that their cohort is worried that AI may replace physicians in the future, while other surveys have reported this to be a non-issue for their study cohort [8, 16]. Gong et al. (2019) found that this anxiety has discouraged students from considering imaging based diagnostic specialities, such as radiology. Our findings are more congruent with a European survey performed by Pinto dos Santos et al. (2021), and did not support anxiety related to physician replacement among medical students. While fears about AI may vary given study cohorts, anxiety regarding the use of AI in a clinical context could be ameliorated by curriculum. Finally, as AI applications progress towards clinical implementation, a lack of understanding could present challenges in effective uptake by physicians [19, 7]. Teaching could address this, in addition to other changing requirements such as the ethical and humanistic role of physicians [3]. Urgent development in medical curriculum is required to accommodate for this growing need [3, 7, 17]. As both this study and previous surveys have confirmed that medical students want AI incorporated into their formal medical curriculum, any such changes should be well received by the undergraduate medical learner population [7, 17].

Previous literature has identified potential objectives for AI teaching, suggesting educational objectives including identifying what technology is appropriate in a specific clinical context, the humanistic and ethical components of AI, and identification of quality improvement applications of AI [3, 7, 20]. This study adds an assessment of existing educational opportunities, preferred formats of AI education by medical students, and potential barriers to uptake. We found that there is no existing formal curriculum about AI at any of the medical schools in Canada. Educational opportunities are similarly limited outside of Canada [21]. Our interviews identified one notable barrier to the inclusion of AI in formal curriculum, being non-AI content taking priority for curricular inclusion. However, as our respondents identified workshops as their preferred learning format, this barrier could be mitigated by use of non-longitudinal educational formats. These would be both more amenable to learners and more easily implemented in a crowded curriculum. Although survey respondents did not believe technical knowledge would be a barrier to uptake of AI, interview participants did express concern about a lack of mathematical or computer science knowledge prevent effective learning about AI. Given the large spectrum of educational backgrounds and experience with technology, it is prudent for medical AI curriculum to restrain from exploring complex technical detail.

The study limitations include non-response and participant bias. While we did receive responses from all medical schools in Canada, our respondent population makes up only ~ 4.5% of the total undergraduate medical student population in Canada [10]. This was in part due to the variable ability of undergraduate medical faculties to support survey dissemination, with some sending the survey as a newsletter, other posting it on the student portal, and other being unable to facilitate distribution. It is likely that participant bias affected study outcomes, and that respondents were more likely to possess interest or knowledge of AI and have a stronger technical understanding than non-respondents. Another limitation is that medical students in later stages of training (e.g. 3rd and 4th year) and male respondents were underrepresented in the survey results. Finally, some aspects of the study design had potential to introduce bias or error. No formal validation process for the survey was employed, outside of the internal pilot to ensure question clarity; this could have reduced face validity or construct validity. We were also unable to comment on how many participants were recruited through each medium, making study reproduction more challenging. Additionally, our survey instrument was lengthy and included questions that were beyond the scope of our research question. The length could have also contributed to participant non-response.

A lack of understanding of AI has been demonstrated over multiple studies, as has the need for curriculum to address AI in medicine. With this survey providing insight into preferred formats of AI education and barriers to AI education, informed development of AI curriculum is possible. We recommend trialling a condensed workshop or lecture, as students reported that they would be most receptive to learning in these formats. Medical education has been traditionally slow to adapt to technological changes, leaving students ill prepared to use technology in clinical practise [22]. However, as policy both in Canada and internationally begins to acknowledge the importance of AI in medicine, financial and institutional support for educational efforts will grow [23]. Future research should seek to develop educational content in the formats indicated above and trial them in a medical student population.

## Conclusion

A lack of educational opportunities about AI in medicine were identified across Canada in the participating medical students. Given medical students overwhelmingly believe that AI is important to the future of medicine and their desire to learn about AI, the development and inclusion of AI in undergraduate medical education should be considered. As AI tools are likely to become widely used in the future, teaching the future generation of physicians about how AI will integrate into clinical workflow will set them up for success, improving the thoughtful implementation of these tools in medical practise and subsequently improving patient care [24].

## Supplementary Information

**Additional file 1.** List of Canadian Medical Schools and status of invitation for both interview and survey.

**Additional file 2.** Complete survey instrument.

**Additional file 3.** Interview questions.

**Additional file 4.** All Likert scale survey responses.

## Data Availability

The datasets used and/or analysed during the current study are available from the corresponding author on reasonable request.

## Abbreviations

AI: Artificial intelligence
ML: Machine learning
CROSS: Consensus-Based Checklist for Reporting of Survey Studies
COREQ: Consolidated Criteria for Reporting Qualitative Research
MS: Medical school (year)
