# Supplementary material for: Exploration of exposure to artificial intelligence in undergraduate medical education: a Canadian cross-sectional mixed-methods study
Source: BMC Med Educ. 2022 Nov 28;22:815. doi: 10.1186/s12909-022-03896-5 (PMC9703803; doi:10.1186/s12909-022-03896-5)
Supplement: Supplementary file 1 — Additional file 1. List of Canadian Medical Schools and status of invitation for both interview and survey. [file 12909_2022_3896_MOESM1_ESM.docx]

**Additional file 1**. List of Canadian Medical Schools and status of invitation for both interview and survey.

| **Medical School** | **Invited to Survey** | **Invited to Interview** |
| --- | --- | --- |
| University of Alberta Faculty of Medicine and Dentistry | Yes | Yes |
| University of Calgary Cumming School of Medicine | Yes | Yes |
| University of British Columbia Faculty of Medicine | Yes | Yes |
| University of Manitoba Max Rady College of Medicine | Yes | Yes |
| Memorial University of Newfoundland Faculty of Medicine | Yes | Yes |
| Dalhousie University Faculty of Medicine | Yes | Yes |
| McMaster University Michael G. DeGroote School of Medicine | Yes | Yes |
| Northern Ontario School of Medicine | Yes | Yes |
| Queen's University School of Medicine | Yes | Yes |
| Western University Schulich School of Medicine and Dentistry | Yes | Yes |
| University of Ottawa Faculty of Medicine | Yes | Yes |
| University of Toronto Faculty of Medicine | Yes | Yes |
| Université Laval Faculté de Médecine | Yes | No |
| McGill University Faculty of Medicine | Yes | Yes |
| Université de Montréal Faculté de Médecine | Yes | No |
| Université de Sherbrooke Faculté de Médecine et des Sciences de la Santé | Yes | No |
| University of Saskatchewan College of Medicine | Yes | Yes |
