# Supplementary material for: Exploration of exposure to artificial intelligence in undergraduate medical education: a Canadian cross-sectional mixed-methods study
Source: BMC Med Educ. 2022 Nov 28;22:815. doi: 10.1186/s12909-022-03896-5 (PMC9703803; doi:10.1186/s12909-022-03896-5)
Supplement: Supplementary file 2 — Additional file 2. Complete survey instrument. [file 12909_2022_3896_MOESM2_ESM.docx]

**Additional file 2**. Complete survey instrument.

**Sections: 1) Screening and Logistics; 2) Demographics; 3) AI in Daily Life; 4) Artificial Intelligence in Medicine; 5) Artificial Intelligence in Medical Education**

*Section 1 – Screening and Logistics*

1. Do you consent to participation in this survey
   1. Yes
   2. No
2. Are you currently a Canadian medical student, or did you graduate a Canadian medical in 2021?
   1. Yes
   2. No
3. Please provide your email address. It will be used to filter out spam responses, enable withdrawal, and contact potential interviews. No identifiable data will ever be published or made publicly available.
4. Do you wish to receive the results of this survey?
   1. Yes
   2. No
5. Would you like to be entered in a draw for 1 of 4 $50 Starbucks gift cards?
   1. Yes
   2. No
6. Would you be willing to provide a short, recorded interview about your responses at a later date?
   1. Yes
   2. No

*Section 2 – Demographics*

1. What is your age?
2. What is your gender?
   1. Male
   2. Female
   3. Non-binary
   4. Other/prefer not to say
3. What year of medical training are you currently in (or have you most recently completed, if responding during the summer)?
   1. 1^st^
   2. 2^nd^
   3. 3^rd^
   4. 4^th^
   5. MSc component of MD MSc
   6. PhD component of MD PhD
4. What medical school do you attend?
   1. University of Alberta Faculty of Medicine and Dentistry
   2. University of Calgary Cumming School of Medicine
   3. University of British Columbia Faculty of Medicine
   4. University of Manitoba Max Rady College of Medicine
   5. Memorial University of Newfoundland Faculty of Medicine
   6. Dalhousie University Faculty of Medicine
   7. McMaster University Michael G. DeGroote School of Medicine
   8. Northern Ontario School of Medicine
   9. Queen's University School of Medicine
   10. Western University Schulich School of Medicine and Dentistry
   11. University of Ottawa Faculty of Medicine
   12. University of Toronto Faculty of Medicine
   13. Université Laval Faculté de Médecine
   14. McGill University Faculty of Medicine
   15. Université de Montréal Faculté de Médecine
   16. Université de Sherbrooke Faculté de Médecine et des Sciences de la Santé
   17. University of Saskatchewan College of Medicine
5. Do you attend medical school at a satellite campus?
   1. Yes
   2. No
6. If you answer “Yes” to the question “Do you attend medical school at a satellite campus?”, which do you attend?
7. What is the highest level of education you achieved before beginning medical education?
   1. High school
   2. College diploma
   3. Bachelor’s degree
   4. Master’s degree
   5. PhD or doctorate
   6. Other/prefer not to say
8. What pre-medical school did you attend?
   1. University of Alberta Faculty of Medicine and Dentistry
   2. University of Calgary Cumming School of Medicine
   3. University of British Columbia Faculty of Medicine
   4. University of Manitoba Max Rady College of Medicine
   5. Memorial University of Newfoundland Faculty of Medicine
   6. Dalhousie University Faculty of Medicine
   7. McMaster University Michael G. DeGroote School of Medicine
   8. Northern Ontario School of Medicine
   9. Queen's University School of Medicine
   10. Western University Schulich School of Medicine and Dentistry
   11. University of Ottawa Faculty of Medicine
   12. University of Toronto Faculty of Medicine
   13. Université Laval Faculté de Médecine
   14. McGill University Faculty of Medicine
   15. Université de Montréal Faculté de Médecine
   16. Université de Sherbrooke Faculté de Médecine et des Sciences de la Santé
   17. University of Saskatchewan College of Medicine
   18. Other (blank)
   19. Prefer not to say
9. Do you have a parent or sibling with an advanced degree (Master’s or higher)
   1. Yes
   2. No
   3. Prefer not to say
10. Do you have a background in mathematics, statistics, or computer science?
    1. Yes
    2. No
    3. Prefer not to say
11. Do you consider yourself to be “tech-savvy”, or have a high degree of technological literacy?
    1. Yes
    2. No
    3. Prefer not to say
12. Which medical residency/specialty are you most interested in or most strongly considering?
    1. Anatomical pathology
    2. Anesthesiology
    3. Anesthesiology – Clinician investigator program
    4. Cardiac surgery
    5. Dermatology
    6. Radiology
    7. Emergency medicine
    8. Family medicine
    9. Family medicine – Integrated care of the elderly
    10. Family medicine – Integrated emergency medicine
    11. General pathology
    12. General surgery
    13. Hematological pathology
    14. Internal medicine
    15. Medical genetics and genomics
    16. Medical microbiology
    17. Neurology
    18. Neurology – Pediatric
    19. Neuropathology
    20. Neurosurgery
    21. Nuclear medicine
    22. Obstetrics and gynecology
    23. Otolaryngology – Head and neck surgery
    24. Pediatrics
    25. Pediatrics – Clinician investigator program
    26. Physical medicine and rehabilitation
    27. Plastic surgery
    28. Plastic surgery – Clinician investigator program
    29. Psychiatry
    30. Psychiatry – Research track
    31. Public health and preventative medicine
    32. Public health and preventative medicine including family medicine
    33. Radiation oncology
    34. Urology
    35. Vascular surgery
    36. Other/prefer not to say

*Section 3 – AI in Daily Life*

1. Many applications we use in daily life use artificial intelligence (e.g. speech-/text-recognition, spam filters, recommendation algorithms, search engines, advertising). Were you aware of this?
   1. Yes
   2. Yes, but I have a very superficial knowledge of these technologies in daily life
   3. No
2. If yes, where have you learned about uses of artificial intelligence in daily life?
   1. Formal education (University, College)
   2. Work experience
   3. Scientific literature
   4. News
   5. Social media
   6. Lectures
   7. Friends/family
   8. Other (blank)
3. Have you learned about artificial intelligence in formal education prior to, or during, medical school?
   1. Yes
   2. No
4. If yes, where did you learn about artificial intelligence in formal education prior to, or during, medical school?
   1. Medical degree curriculum
   2. Optional course offered by a medical school
   3. Online course
   4. Research project
   5. As part of an advanced degreed (Master’s, PhD etc.)
   6. Undergraduate degree
   7. Other (blank)
5. Do you feel that a lack of understanding of programming or mathematics is a barrier in your understanding of artificial intelligence methods and applications?
   1. Yes
   2. No

*Section 4 – Artificial Intelligence in Medicine*

Definitions for reference:

Artificial intelligence (AI): The use of computers and technology to simulate intelligent behavior and critical thinking comparable to a human being. AI applications can emulate processes characteristic of humans, such as the ability to reason, discover meaning, generalize, or learn with experience.

Machine learning (ML): Computer software that can learn autonomously using examples. In other words, the process by which a computer is able to improve its own performance by continuously incorporating new data into an existing model.

Neural network: A program inspired by the neural network in the brain. Artificial neural networks can perform cognitive functions such as problem solving and machine learning.

1. Are you aware that artificial intelligence, machine learning, neural networks, and deep learning techniques are currently utilized in medicine?
   1. Yes
   2. Yes, but I have a very superficial knowledge of these technologies in medicine
   3. No
2. If yes, what sources did you learn about artificial intelligence, machine learning, neural networks, and deep learning techniques are currently utilized in medicine?
   1. Formal education during medical school
   2. Formal education in advanced degree (Master’s, PhD etc.)
   3. Formal education in undergraduate degree
   4. My peers (e.g. other medical students or other students in the field)
   5. My friends (friends who are not in healthcare)
   6. My mentors/teachers (those who are involved in your education or training)
   7. Published research papers
   8. News, media
   9. Movies and TV series
   10. Other (blank)
3. According to the scale provided, how accurate are the following statements? Please score the following statements according to the scale: (1) Strongly disagree; (2) Disagree; (3) Neutral; (4) Agree; (5) Strongly agree; (6) Not applicable/no comment. [Likert scale]
   1. I could describe what artificial intelligence, machine learning, neural networks, and/or deep learning are.
   2. I could provide examples of artificial intelligence, machine learning, neural networks, and/or deep learning applications in medicine.
   3. I understand artificial intelligence research methods.
   4. Artificial intelligence applications are commonly used in medicine.
   5. Artificial intelligence applications have improved medicine.
   6. Artificial intelligence applications in medicine will become common in the future.
   7. Artificial intelligence will improve medicine in the future.
   8. Artificial intelligence will revolutionize medicine in the future.
4. According to the scale provided, how accurate are the following statements? Please score the following statements according to the scale: (1) Strongly disagree; (2) Disagree; (3) Neutral; (4) Agree; (5) Strongly agree; (6) Not applicable/no comment. [Likert scale]
   1. Artificial intelligence applications will be cost-effective.
   2. Artificial intelligence will cause more benefits than harm.
   3. Artificial intelligence will optimize physician’s work.
   4. There is a lot of hype surrounding artificial intelligence in medicine, but it will soon be over.
   5. In the future some or all physicians will be replaced by AI.
   6. The development of artificial intelligence in medicine frightens me.
   7. Artificial intelligence will never make the human physician expendable.
   8. The medical specialty or discipline that I am interested in pursuing will be particularly affected by artificial intelligence.
5. If you associate artificial intelligence applications with a specific discipline in medicine, which ones (fill as many as appropriate)?
   1. Anatomical pathology
   2. Anesthesiology
   3. Cardiac surgery
   4. Dermatology
   5. Radiology
   6. Emergency medicine
   7. Family medicine
   8. Family medicine – Integrated care of the elderly
   9. Family medicine – Integrated emergency medicine
   10. General pathology
   11. General surgery
   12. Hematological pathology
   13. Internal medicine
   14. Medical genetics and genomics
   15. Medical microbiology
   16. Neurology
   17. Neurology – Pediatric
   18. Neuropathology
   19. Neurosurgery
   20. Nuclear medicine
   21. Obstetrics and gynecology
   22. Otolaryngology – Head and neck surgery
   23. Pediatrics
   24. Physical medicine and rehabilitation
   25. Plastic surgery
   26. Psychiatry
   27. Public health and preventative medicine
   28. Public health and preventative medicine including family medicine
   29. Radiation oncology
   30. Urology
   31. Vascular surgery
   32. Other/prefer not to say

*Section 5 – Artificial Intelligence in Medical Education*

1. According to the scale provided, how accurate are the following statements? Please score the following statements according to the scale: (1) Strongly disagree; (2) Disagree; (3) Neutral; (4) Agree; (5) Strongly agree; (6) Not applicable/no comment. [Likert scale]
   1. I will need to understand artificial intelligence during my medical career.
   2. I will use artificial intelligence applications during my medical career.
   3. Artificial intelligence should be a formally taught topic in medical education.
   4. I have received training in the use of artificial intelligence applications in medicine in formal curriculum (classes, lectures, small groups).
   5. I believe that I will receive training in the use of artificial intelligence applications in medicine in formal curriculum (classes, lectures, small groups) in the future.
   6. I have received training in the use of artificial intelligence in medicine externally (independently attended talks, conferences, lectures, workshops).
2. According to the scale provided, how accurate are the following statements? Please score the following statements according to the scale: (1) Strongly disagree; (2) Disagree; (3) Neutral; (4) Agree; (5) Strongly agree; (6) Not applicable/no comment. [Likert scale]
   1. I have received training in the use of artificial intelligence in medicine through research or work experiences.
   2. I have independently educated myself about artificial intelligence in medicine (Google, PubMed, literature search, news articles).
   3. I feel like my learning opportunities about artificial intelligence in medicine have been adequate.
   4. I think that it is important that I better study artificial intelligence in medicine.
   5. I feel that my understanding of programming or mathematics is a barrier to my understanding of artificial intelligence in medicine.
   6. Given the chance, I would like to learn more about artificial intelligence.
3. My preferred format for learning about artificial intelligence and machine learning in medicine are (fill as many as appropriate)
   1. Lectures
   2. Workshops
   3. Conferences
   4. Extracurricular activities
   5. Collaborative activities with other departments (mathematics, computer science)
   6. Other (blank)
