# Supplementary material for: Exploration of exposure to artificial intelligence in undergraduate medical education: a Canadian cross-sectional mixed-methods study
Source: BMC Med Educ. 2022 Nov 28;22:815. doi: 10.1186/s12909-022-03896-5 (PMC9703803; doi:10.1186/s12909-022-03896-5)
Supplement: Supplementary file 3 — Additional file 3. Interview questions. [file 12909_2022_3896_MOESM3_ESM.docx]

**Additional file 3**. Interview questions.

1. Could you tell us your age, academic background, which year of medical school you are currently in (or have most recently completed)?
2. What do you know about artificial intelligence and/or machine learning in medicine?
3. Do you think it is important that you learn about artificial intelligence and/or machine learning in medical school?
4. Do you think AI will be important in your practise as a physician?
5. What opportunities have you had to learn about artificial intelligence and/or machine learning in medicine in either formal curriculum or outside of formal medical curriculum?
6. What learning opportunities to learn about artificial intelligence and/or machine learning in medicine would you like to have in the future, or do you wish you had previously?
7. [open-ended] Is there anything else you would like to share about artificial intelligence and/or machine learning in medicine or the education of these topics?
