# Supplementary material for: Exploration of exposure to artificial intelligence in undergraduate medical education: a Canadian cross-sectional mixed-methods study
Source: BMC Med Educ. 2022 Nov 28;22:815. doi: 10.1186/s12909-022-03896-5 (PMC9703803; doi:10.1186/s12909-022-03896-5)
Supplement: Supplementary file 4 — Additional file 4. All Likert scale survey responses. [file 12909_2022_3896_MOESM4_ESM.docx]

**Additional file 4.** All Likert scale survey responses.

| **Question Item** | **N** | **Likert scale response; n (%)** | | | | | |
| --- | --- | --- | --- | --- | --- | --- | --- |
|  |  | **Strongly agree** | **Agree** | **Neutral** | **Disagree** | **Strongly disagree** | **Consensus** |
| I could describe what artificial intelligence, machine learning, neural networks, and/or deep learning are. | 472 | 34 (7.2) | 148 (31.4) | 93 (19.7) | 156 (33.1) | 41 (8.7) | Mixed (38.6% agree, 41.7% disagree) |
| I could provide examples of artificial intelligence, machine learning, neural networks, and/or deep learning applications in medicine. | 463 | 40 (8.6) | 176 (38.0) | 71 (15.3) | 138 (29.8) | 38 (8.2) | Mixed (46.7% agree, 38.0% disagree) |
| I understand artificial intelligence research methods. | 463 | 18 (3.9) | 70 (15.1) | 83 (17.9) | 195 (42.1) | 97 (21.0) | Disagree (63.1%) |
| Artificial intelligence applications are commonly used in medicine. | 457 | 54 (11.8) | 217 (47.5) | 138 (30.2) | 45 (9.8) | 3 (0.7) | Agree (59.3%) |
| Artificial intelligence applications have improved medicine. | 456 | 102 (22.4) | 234 (51.3) | 110 (24.1) | 8 (1.8) | 2 (0.4) | Agree (73.7%) |
| Artificial intelligence applications in medicine will become common in the future. | 453 | 242 (53.4) | 182 (40.2) | 27 (6.0) | 2 (0.4) | 0 (0.0) | Agree (93.6%) |
| Artificial intelligence will improve medicine in the future. | 455 | 172 (37.8) | 210 (46.2) | 65 (14.3) | 6 (1.3) | 2 (0.4) | Agree (84.0%) |
| Artificial intelligence will revolutionize medicine in the future. | 454 | 150 (33.0) | 186 (41.0) | 93 (20.5) | 23 (5.1) | 2 (0.4) | Agree (74.0%) |
| Artificial intelligence applications will be cost-effective. | 457 | 95 (20.8) | 199 (43.5) | 133 (29.1) | 25 (5.5) | 5 (1.1) | Agree (64.3%) |
| Artificial intelligence will cause more benefits than harm. | 454 | 58 (12.8) | 198 (43.6) | 155 (19.6) | 41 (9.0) | 2 (0.4) | Agree (50.4%) |
| Artificial intelligence will optimize physician’s work. | 455 | 96 (21.1) | 256 (56.3) | 89 (19.6) | 11 (2.4) | 3 (0.7) | Agree (77.4%) |
| There is a lot of hype surrounding artificial intelligence in medicine but it will soon be over. | 448 | 5 (1.1) | 23 (5.1) | 76 (17.0) | 297 (66.3) | 47 (10.5) | Disagree (76.8%) |
| In the future some or all physicians will be replaced by AI. | 461 | 13 (2.8) | 81 (17.6) | 65 (14.1) | 181 (39.3) | 121 (26.2) | Disagree (65.5%) |
| The development of artificial intelligence in medicine frightens me. | 464 | 18 (3.9) | 101 (21.8) | 101 (21.8) | 182 (39.2) | 62 (13.4) | Disagree (52.6%) |
| Artificial intelligence will never make the human physician expendable. | 455 | 137 (30.1) | 170 (37.4) | 82 (18.0) | 50 (11.0) | 16 (3.5) | Agree (67.5%) |
| The medical specialty or discipline that I am interested in pursuing will be particularly affected by artificial intelligence. | 441 | 37 (8.4) | 103 (23.4) | 146 (33.1) | 127 (28.8) | 28 (6.3) | Mixed (31.7% agree, 35.1% disagree) |
| I will need to understand artificial intelligence during my medical career. | 463 | 104 (22.5) | 212 (45.8) | 92 (19.9) | 51 (11.0) | 4 (0.9) | Agree (68.3%) |
| I will use artificial intelligence applications during my medical career. | 457 | 110 (41.1) | 223 (48.8) | 109 (23.9) | 15 (3.3) | 0 (0.0) | Agree (72.9%) |
| Artificial intelligence should be a formally taught topic in medical education. | 455 | 99 (21.8) | 205 (45.1) | 103 (22.6) | 43 (9.5) | 5 (1.1) | Agree (66.8%) |
| I have received training in the use of artificial intelligence applications in medicine in formal curriculum (classes, lectures, small groups). | 446 | 11 (2.5) | 18 (4.0) | 40 (9.0) | 196 (43.9) | 181 (40.6) | Disagree (84.5%) |
| I believe that I will receive training in the use of artificial intelligence applications in medicine in formal curriculum (classes, lectures, small groups) in the future. | 449 | 11 (2.4) | 70 (15.6) | 103 (22.9) | 196 (43.7) | 69 (15.4) | Disagree (59.0%) |
| I have received training in the use of artificial intelligence in medicine externally (independently attended talks, conferences, lectures, workshops). | 445 | 33 (7.4) | 85 (19.1) | 35 (7.1) | 191 (42.9) | 101 (22.7) | Disagree (65.6%) |
| I have received training in the use of artificial intelligence in medicine through research or work experiences. | 453 | 34 (7.5) | 63 (13.9) | 37 (8.2) | 197 (43.5) | 122 (26.9) | Disagree (70.4%) |
| I have independently educated myself about artificial intelligence in medicine (Google, PubMed, literature search, news articles). | 458 | 53 (11.6) | 139 (30.3) | 72 (15.7) | 135 (29.5) | 59 (12.9) | Mixed (41.9% agree, 42.4% disagree) |
| I feel like my learning opportunities about artificial intelligence in medicine have been adequate. | 450 | 11 (2.4) | 31 (6.9) | 77 (17.1) | 244 (54.2) | 87 (19.3) | Disagree (73.6%) |
| I think that it is important that I better study artificial intelligence in medicine. | 459 | 70 (15.3) | 214 (46.6) | 118 (35.7) | 47 (10.2) | 10 (2.2) | Agree (61.9%) |
| I feel that my understanding of programming or mathematics is a barrier to my understanding of artificial intelligence in medicine. | 453 | 70 (15.5) | 141 (31.1) | 86 (19.0) | 121 (26.7) | 35 (7.7) | Agree (46.6%) |
| Given the chance, I would like to learn more about artificial intelligence. | 462 | 150 (32.5) | 210 (45.5) | 76 (16.5) | 20 (4.3) | 6 (1.3) | Agree (77.9%) |
